# Deficient amygdala–prefrontal intrinsic connectivity after effortful emotion regulation in borderline personality disorder

**DOI:** 10.1007/s00406-016-0760-z

**Published:** 2016-12-30

**Authors:** Blazej M. Baczkowski, Linda van Zutphen, Nicolette Siep, Gitta A. Jacob, Gregor Domes, Simon Maier, Andreas Sprenger, Alena Senft, Bastian Willenborg, Oliver Tüscher, Arnoud Arntz, Vincent van de Ven

**Affiliations:** 1 Department of Clinical Psychological Science, Faculty of Psychology and Neuroscience, Maastricht University, P.O. Box 616, 6200 MD Maastricht, The Netherlands; 2 Max Planck Research Group for Neuroanatomy & Connectivity, Max Planck Institute for Human Cognitive and Brain Sciences, Leipzig, Germany; 3 Department of Clinical Psychology and Psychotherapy, University of Freiburg, Freiburg, Germany; 4 Department of Psychology, Laboratory for Biological and Personality Psychology, University of Freiburg, Freiburg, Germany; 5 Freiburg Brain Imaging Center, University Medical Center, University of Freiburg, Freiburg, Germany; 6 Department of Biological and Clinical Psychology, University of Trier, Trier, Germany; 7 Department of Psychiatry and Psychotherapy, Medical Center, University of Freiburg, Freiburg, Germany; 8 Departments of Neurology and Psychology, University of Lübeck, Lübeck, Germany; 9 Department of Psychiatry and Psychotherapy, University of Lübeck, Lübeck, Germany; 10 Department of Psychiatry and Psychotherapy, University Medical Center Mainz, Mainz, Germany; 11 Department of Clinical Psychology, University of Amsterdam, Amsterdam, The Netherlands; 12 Department of Cognitive Neuroscience, Maastricht University, Maastricht, The Netherlands

**Keywords:** BPD, Emotion regulation, Amygdala, Functional connectivity, Resting-state fMRI

## Abstract

Emotion instability in borderline personality disorder (BPD) has been associated with an impaired fronto-limbic inhibitory network. However, functional connectivity (FC) underlying altered emotion regulation in BPD has yet to be established. Here, we used resting-state fMRI to investigate enduring effects of effortful emotion regulation on the amygdala intrinsic FC in BPD. In this multicenter study, resting-state fMRI was acquired before and after an emotion regulation task in 48 BPD patients and 39 non-patient comparison individuals. The bilateral amygdalae were used as a seed in the whole-brain FC analysis and two-way mixed ANOVA to test whether BPD patients exhibited weaker post-task increase in the amygdala intrinsic FC with the prefrontal cortex (PFC), compared to non-patients. Subsequently, we explored whether the results are common for personality disorders characterized by emotional problems, using additional data of 21 cluster-C personality disorder patients. In contrast to non-patients, BPD patients failed to show increased post-task amygdala resting-state FC with the medial, dorsolateral, ventrolateral PFC, and superior temporal gyrus, but surprisingly exhibited decreased FC with the posterior cingulate cortex and increased FC with the superior parietal lobule. In BPD patients, the emotion regulation task failed to increase resting-state amygdala FC with brain regions essential for effortful emotion regulation, which suggests: (a) altered cognitive control typically used to indirectly alleviate distress by reinterpreting the meaning of emotional stimuli; (b) impaired direct regulation of emotional responses, which might be common for personality disorders; (c) avoidance of self-related appraisals induced by social emotional stimuli.

**Electronic supplementary material:**

The online version of this article (doi:10.1007/s00406-016-0760-z) contains supplementary material, which is available to authorized users.

## Introduction

Borderline personality disorder (BPD) is a severe psychiatric disorder with the prevalence in the general population estimated at approximately 1–3% [1, 2]. Patients with BPD are characterized by a pervasive pattern of instability in self-image, interpersonal relationships, affect, and impulsive behavior [DSM-V, 3]. Leading conceptualizations posit that BPD is best understood as a disorder of emotion regulation [4–6].

Functional neuroimaging studies highlight that clinically well-observed BPD features of emotion dysregulation—emotional hypersensitivity and intense emotional reactions—are due to increased limbic and diminished prefrontal activity [for meta-analysis and reviews: [7–9]], which suggests an impaired fronto-limbic inhibitory network. Altered subcortical-cortical functional connectivity (FC) of BPD patients has been identified with fMRI during experimental induction of negative emotions [10–12] and in task-free resting-state [e.g., 13]. Compared to non-patients (NPC), BPD patients exhibit increased amygdala FC with the subgenual anterior cingulate cortex (ACC) when viewing fearful faces [10] and with the ventromedial prefrontal cortex (vmPFC) when exposed to threat [11]. In BPD, induction of pain together with emotionally arousing pictures is associated with enhanced negative FC of the amygdala with the medial and dorsolateral PFC [12]. Furthermore, BPD patients exhibit stronger FC of both the amygdala and dorsal ACC with the dorsomedial prefrontal cortex (dmPFC) during emotional distraction [14]. Resting-state fMRI studies showed that emotional hypersensitivity of BPD patients is associated with hyperconnectivity within the salience network [15], i.e., between the amygdala and bilateral insula together with dorsal ACC [13, 16–19], while their impaired control over emotional reactions is associated mostly with diminished intrinsic connectivity between the central executive fronto-parietal regions and salience network [16, 17]. Both Doll et al. [17] and Wolf et al. [19] reported aberrant FC between the regions of the central executive fronto-parietal network at rest. Taken together, BPD patients show altered FC within the amygdala–PFC network when confronted with negative emotions, which is assumed to contribute to their affective instability and in the long-term shapes the organization of their resting-state networks.

However, brain FC associated with emotion regulation in BPD patients has yet to be established. Despite long-term stability of resting-state networks, resting-state functional connectivity (rsFC) exhibits substantial variations at the timescales of minutes [for review, [20]] and has been successfully used to investigate post-task changes in FC induced by cortical activation during behavioral manipulations [e.g., 21, 22], and stress exposure [23, 24] or memory processes [25, 26]. Hence, resting-state fMRI can be used to investigate whether effortful emotion regulation induces enduring aberrant patterns of FC in BPD patients.

In the present international multicenter study, resting-state fMRI data were acquired before and after an emotion regulation task in 48 BPD patients and 39 NPC. We used an amygdala seed-based approach, since the amygdala is involved in emotion generation and its activity can be modulated by the prefrontal regions during effortful emotion regulation [27–29]. The increased coupling of the amygdala with the prefrontal regions is typically present during active regulation and consequently may alleviate emotional distress [30, 31]. We hypothesized that in NPC effortful emotion regulation would lead to increased post-task amygdala rsFC with these prefrontal regions, whereas BPD patients would exhibit weaker increases. Subsequently, we explored whether the results are specific for BPD or common for personality disorders, including 21 cluster-C personality disorder (CPD) patients. BPD patients are often additionally diagnosed with one of the CPDs [32], and emotional problems are common for both disorders, which, however, might be associated with different neurocognitive mechanisms [33].

## Methods and materials

### Participants

Sixty-two BPD patients, 48 NPC, and 31 CPD patients were recruited from two sites in the Netherlands (Maastricht, Heerlen) and three sites in Germany (Freiburg, Lübeck, Hamburg). BPD and CPD patients were recruited from mental health clinics at local sites. NPC were recruited among the general population at each site. Participants had to be hetero- or bisexual females, aged 18–65, and sufficient understanding of the language of the local sites. Participants who did not fulfill the scanning or clinical criteria were excluded, and the final sample comprised 48 BPD patients, 39 NPC, and 21 CPD patients. Detailed description of additional measures including: BPD Severity Index [BPDSI; 34–36], Brief Symptom Inventory [BSI; 37], BPD checklist [38] and Interview for Trauma Events in Childhood [ITEC; 39], participant recruitment and exclusion procedure, is provided in the supplementary material.

Demographics and clinical measures of the sample are presented in Table 1. The groups did not significantly differ for age, handedness preference, and IQ. After complete description of the study, all participants provided written informed consent and received financial remuneration for their participation. The study was approved by the local medical ethical committees [40].

**Table 1:** Descriptive statistics of the three groups: borderline personality disorder (BPD), non-patient comparison individuals (NPC), and cluster-C personality disorder (CPD)

	BPD (*n* = 48)		NPC (*n* = 39)		CPD (*n* = 21)		Test statistics	
	Mean	SD	Mean	SD	Mean	SD	*F*	*p*
Age (years)	30.79	9.21	28.67	10.70	31.48	11.80	0.67	0.512
Estimated IQ^a^	96.88	10.08	100.73	11.38	98.45	9.26	1.45	0.239^b^
Brief symptom inventory	1.68	0.55	0.13	0.13	1.11	0.43	142.19	<0.001^c^
BPD checklist	118.44	25.58	50.68	5.21	74.80	17.31	135.21	<0.001^c^
Interview traumatic events childhood							8.85	<0.001^d^
Sexual abuse	9.44	8.85	0.13	0.42	2.26	5.43	21.21	<0.001
Physical abuse	17.15	12.40	1.69	3.61	7.00	10.95	23.68	<0.001
Emotional abuse	20.35	8.93	2.47	3.32	13.06	8.49	54.52	<0.001
Emotional neglect	11.28	6.99	0.82	2.07	6.14	6.55	31.66	<0.001
Physical neglect	10.68	9.42	0.88	2.82	4.35	6.90	17.67	<0.001
Dissociation^e^							5.00	<0.01^f^
Prior scanning	19.81	19.41	5.18	6.60	6.37	6.98	13.55	<0.001^g^
Post scanning	31.87	26.85	6.40	7.81	15.36	20.60	16.02	<0.001^g^
	%	n	%	n	%	n	χ^2^	*p*
Education level^h^							7.70	0.02^i^
Level 1	22.9	11	17.9	7	14.3	3		
Level 2	14.6	7	5.1	2	19.0	4		
Level 3	27.1	13	10.3	4	28.6	6		
Level 4	4.2	2	5.1	2	14.3	3		
Level 5	25.0	12	43.6	17	14.3	3		
Level 6	6.3	3	17.9	7	9.5	2		
Handedness							6.00	0.20^j^
Left	8	4	5	2	–	–		
Right	86	41	95	37	100	21		
Mixed	6	3	–	–	–	–		
Axis I disorders								
Major depressive disorder	87.5	42			61.9	13	5.92	0.02
Dysthymic	8.3	4			4.8	1	0.28	0.60
Bipolar type II	2.1	1			–	–	0.44	0.51
Generalized anxiety disorder	4.2	2			4.8	1	0.12	0.91
Panic disorder with agoraphobia	12.5	6			–	–	2.88	0.09
Panic disorder	12.5	6			14.3	3	0.41	0.84
Agoraphobia	8.3	4			–	–	1.86	0.17
Specific phobia	18.8	9			4.8	1	2.31	0.13
Social phobia	31.2	15			23.8	5	0.39	0.53
Obsessive compulsive disorder	14.6	7			9.5	2	0.33	0.57
Posttraumatic stress disorder	35.4	17			14.3	3	3.17	0.08
Somatoform disorder	10.4	5			19.0	4	0.96	0.33
Eating disorders	35.4	17			33.3	7	0.03	0.87
Substance abuse	43.8	21			4.8	1	10.23	0.001
Intermitted explosive disorder	2.1	1			–	–	0.44	0.51
Axis II disorders								
Avoidant PD	43.8	21			71.4	15	4.49	0.03
Dependent PD	14.6	7			9.5	2	0.33	0.57
Obsessive compulsive PD	20.8	10			33.3	7	1.23	0.27
Passive aggressive PD	6.2	3			–	–	1.37	0.24
Depressive PD	25.0	12			9.5	2	2.16	0.14
Paranoid PD	29.2	14			–	–	7.68	<0.01
Schizotypal PD	2.1	1			–	–	0.44	0.51
Schizoid PD	2.1	1			–	–	0.44	0.51
Medication								
Antidepressants	64.6	31			36.4	8	4.17	0.04
Antipsychotics	10.4	5			–	–	2.36	0.13
Hypnotics	4.2	2			–	–	0.90	0.34
Mood stabilizers	2.1	1			–	–	0.44	0.51

^a^Assessed with four subtasks of the WAIS (i.e., vocabulary, similarities, block design, and matrix reasoning)

^b^Data of one NPC was not available

^c^All three groups significantly differed from each other (*p* < 0.001). Data of two NPC and one CPD patient were not available

^d^MANOVA and ANOVAs showed significant group differences over the childhood traumatic events. BPD patients reported more traumatic events than either CPD patients or NPC with respect to sexual abuse, physical abuse, emotional abuse, emotional neglect, and physical neglect (all *p*s < 0.01). CPD patients reported more traumatic events than NPC with respect to emotional abuse and emotional neglect (all *p*s < 0.01). Data from seven NPC and one CPD patient were not available

^e^Measured with the four somatic dissociation items of the Dissociation-Tension-Scale (i.e., derealization and change in perception of one’s body, hearing, and pain)

^f^Two-way mixed-design ANOVA showed a significant group × time interaction. In BPD patients, the increase in reported dissociative states was significantly larger than in NPC (*p* = 0.001). Data from five BPD patients, two NPC, and one CPD patient were not available

^g^ANOVA showed a significant group effect over dissociation. BPD patients showed increased level of dissociation as compared to NPC (*p* < 0.001) as well as to CPD group (*p* < 0.001)

^h^Level of education of both the Dutch and German educational systems was translated into the International Standard Classification of Education (ISCED), and in the current study, six levels of education were divided ranging from lower secondary school to Master’s degree

^i^Value is based on the Kruskal–Wallis test. Data of one NPC was not available

^j^Value is based on the Chi-square goodness-of-fit test

### Study design

Two six-minute resting-state runs, during which participants were instructed to lie still, relax, and keep their eyes open, were part of a larger study investigating emotion dysregulation in BPD [van Zutphen et al., submitted]. Resting-state runs were acquired before and after an emotion regulation task, which was an adapted version of previously published emotional regulation paradigm [41, 42]. In this task, participants were presented with negative, positive, erotic, and neutral pictures and instructed to either attend to the picture and respond naturally without altering their emotional state (passive viewing condition) [42], or to regulate their emotional state by realizing that they are safe (regulation condition), a technique inspired by schema therapy [43]. Since BPD patients are particularly responsive to interpersonal stimuli [41], only pictures with a social content (i.e., one person emotionally relating to the viewer or two or more persons in interaction) were selected. Each trial consisted of a 2 s visual instruction to “look” or “realize being safe”, 8 s presentation of the pictures while implementing the instruction, 4 s rating period and variable 5–6.5 s fixation period (for schematic overview Fig. S1). During the rating period, participants assessed their momentary emotional state on a horizontal visual analogue scale (−100 to 100 mm). The order of the conditions and stimulus categories were equally divided and presented in a pseudo-randomized order. The task consisted of 96 trials divided into four runs of 24 trials each. The time between the two resting-state scans was about 45 min (two runs of the task, then an anatomical scan, next two more runs of the task). The experimental manipulation successfully affected the subjective ratings and brain activation [van Zutphen et al., submitted]. After the MRI data acquisition, participants evaluated the arousal and valence of each picture presented in the task, using the Self-Assessment Manikin [44]. Furthermore, participants were asked to fill out an exit questionnaire, in which they stated their compliance to the study instructions. Finally, prior to the scanning session and immediately afterward participants rated their level of dissociative experiences with the Dissociation-Tension-Scale [45] as well as the level of anxiety and nervousness.

### FMRI data acquisition and statistical analyses

Structural and functional MRI data were acquired with 3-Tesla scanners at each site. Participants were scanned in head first supine position. Head movements were minimized using foam paddings. Functional images were taken with a T2*-weighted echo planar imaging (EPI) sequence with the following parameters: 180 volumes, TR = 2000 ms, TE = 27 ms, flip angle = 90°, FoV = 192 × 192 mm, voxel size = 3 × 3 × 3 mm, and matrix = 64 × 64. One volume in Maastricht consisted of 32, and in Freiburg and Lübeck of 34, interleaved measured axial slices. The T2*-weighted slices were optimized with a negative tilt of 30°, to minimize susceptibility and distortion artifacts within the amygdala [46] in Maastricht and Freiburg. Anatomical images were acquired with high-resolution T1-weighted sequence with the following parameters: TR = 2250 ms, TE = 2.6 ms, flip angle = 9°, FoV = 256 × 256 mm, voxel size 1 × 1 × 1 mm. In total, 192 slices were obtained in Maastricht, 160 in Freiburg, and 170 in Lübeck. Scanner specifications and preprocessing steps are described in the supplementary material.

The statistical analyses were performed using BrainVoyager 2.8 (Brain Innovation, Maastricht, The Netherlands), SPSS Statistics 21 (IBM Corp, NY), NeuroElf (MR imaging analysis toolbox, www.neuroelf.net), and custom routines in MATLAB (Mathworks, Natick, MA). An amygdala seed-based whole-brain correlation approach was used. The masks for left and right amygdala were obtained by applying a sphere of 5 mm radius around the coordinates (Tal: ±22, −6, −14) which is similar to that used in the previous rsFC studies in NPC [24] and in BPD [13]. The amygdala seeds of the left and right hemisphere are depicted in Fig. S2.

Functional connectivity was estimated with the Pearson *r* correlation coefficient. The time courses of the two amygdala seeds were extracted, due to high correlations between the left and right seed averaged [13, 14], and then correlated with the time course of all other voxels in the brain on the individual level. The resulting *r* values were converted to z-scores using Fisher’s transformation in order to increase normality of the distribution. The obtained z-scores were then entered into a second-level analysis. We attempted to equalize the scanner parameters across sites, but at the local sites not everything could be translated exactly the same; therefore, a discrete factor representing site was entered into a regression analysis to estimate the effect of site on the amygdala rsFC. The resulting residual amygdala rsFC data were used in the subsequent random effects analysis, thereby accounting for effects of site. Multiple comparisons across space were FWE corrected on a cluster level, using Monte Carlo simulation (1000 iterations) based on the smoothness of a statistical map [47, 48]. Statistical maps were first thresholded at *p* = 0.01 and then corrected at the 3D cluster level at *p* = 0.05.

To determine whether the emotion regulation task led to differential changes in amygdala rsFC in BPD compared to NPC, we calculated a two-way mixed ANOVA and analyzed the time (before vs. after the task) × group (BPD vs. NPC) interaction. To avoid detection of “spurious” clusters of interaction, we restricted the interpretation of the interaction F-map in the following way. First, in order to indicate whether there were any baseline group differences in the amygdala rsFC before the emotion regulation task, we performed two-tailed independent sample *t* test with the contrast BPD versus NPC. Second, to indicate whether there were significant changes in the rsFC of the amygdala after the task, we performed two-tailed paired *t* test in each group with the contrast resting-state after versus before the task. The t-map represents a putative change in the amygdala rsFC for each group, respectively. Following, we used a conjunction analysis of the two t-maps to determine whether there was any common network of the post-task change for both groups. With these restrictions, we limited our interpretation to only those interaction effects that were based on an effect of the task in at least one group regardless any potential baseline group differences. The individual average z-score (the strength of the rsFC) was extracted from the remaining clusters and exported to SPSS for analysis of simple effects to indicate the direction of a significant connectivity change in each group. Finally, each cluster was investigated post hoc for the confounding effect of medication within the BPD group.

Further, we performed correlation analyses within the BPD group to test whether the differences in severity of BPD pathology and traumatic events in childhood were associated with the magnitude of change in the amygdala rsFC network representing differential post-task change in BPD compared to NPC. BPD pathology was indicated by individual scores of the BPDSI, BSI, BPD checklist, and the baseline level of dissociation measured with a four-item Dissociation-Tension-Scale. The severity of childhood abuse and neglect was expressed by a summation of the ITEC subscales. In addition, we assessed whether the change (increase) in the level of dissociation before versus after scanning is associated with change in the amygdala rsFC to explore the impact of more state-dependent BPD characteristics. These scores were correlated with the magnitude of change in amygdala rsFC with the clusters retrieved from the group × time interaction. The magnitude was indicated by subtracting the z-scores of the resting-state before from the resting-state after the task, indicating the higher the z-score, the more increase in the rsFC after the task. Each correlational analysis was family-wise error (FWE) corrected by Bonferroni procedure.

Lastly, we explored whether the results of altered changes in the amygdala rsFC after the emotion regulation task are specific for BPD or common with CPD. We performed a two-way mixed ANOVA in BPD versus CPD and analyzed the time × group interaction on the individual z-scores. We restricted the ANOVA model to the brain regions that previously indicated the significant interaction effect between BPD and NPC. The analysis was FWE corrected by Bonferroni procedure.

## Results

### Behavioral results

To determine whether each group implemented the task instructions and regulated their emotional state, the ratings of emotional state obtained immediately after the passive viewing or regulation condition across stimulus categories were compared between BPD and NPC. Both groups implemented the task instructions, as indicated by the significant change in their behavioral performance of the regulation condition, compared to the passive viewing condition [NPC: M = 16.67, SD = 41.81, *t*(37) = 2.46, *p* = 0.02; BPD: M = 11.90, SD = 31.41, *t*(46) = 2.60, *p* = 0.01]. Additionally, the condition × group interaction was not significant (*p* = 0.55), indicating that both groups regulated their emotional state to a similar extent between the two resting-state runs.

### Group differences in functional connectivity at baseline

A common network of the intrinsic amygdala connectivity was obtained in both BPD and NPC before and after the emotion regulation task (Fig. S3a), which was identified as similar to patterns previously described in the literature [e.g., 49].

**Fig. 1:**
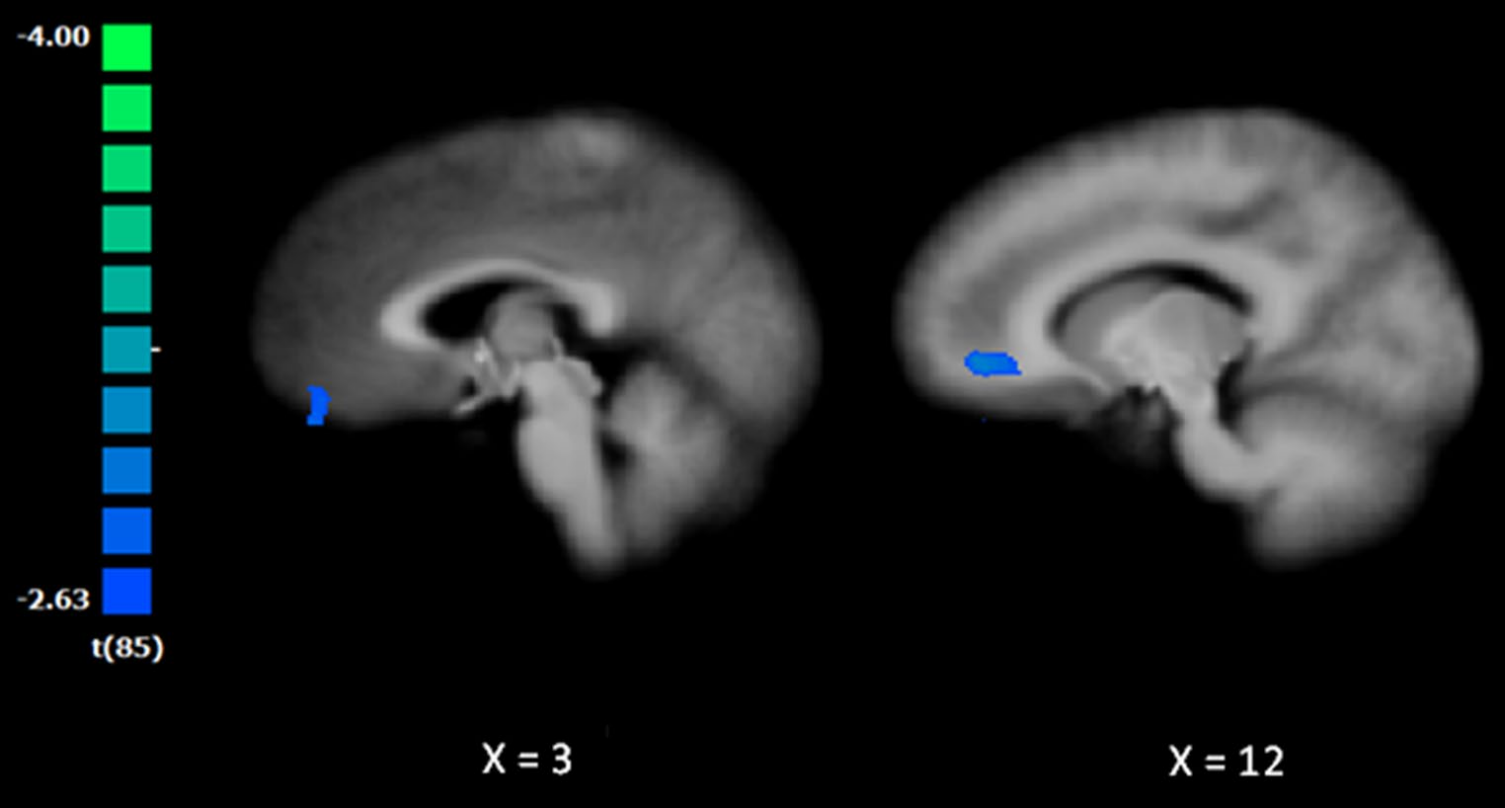
Baseline differences in the functional connectivity between BPD and NPC. The t-map was corrected at the cluster level (*p* = 0.05; *k* = 675 mm^3^) and overlaid on an anatomical image averaged over all participants in the Talairach standard space, according to the radiological convention. The cold colors indicate weaker resting-state functional connectivity of the amygdala in the BPD patients compared to the NPC group

At baseline, BPD patients exhibited decreased amygdala rsFC with a cluster comprising the right ventral ACC and right orbitofrontal cortex [peak coordinates Tal: 11, 46, 0; *t*(85) = 3.89; Fig. 1]. No significant differences were obtained for the cluster between BPD patients who were medicated versus non-medicated (*p* = 0.85).

### Change in functional connectivity after the emotion regulation task

**Fig. 2:**
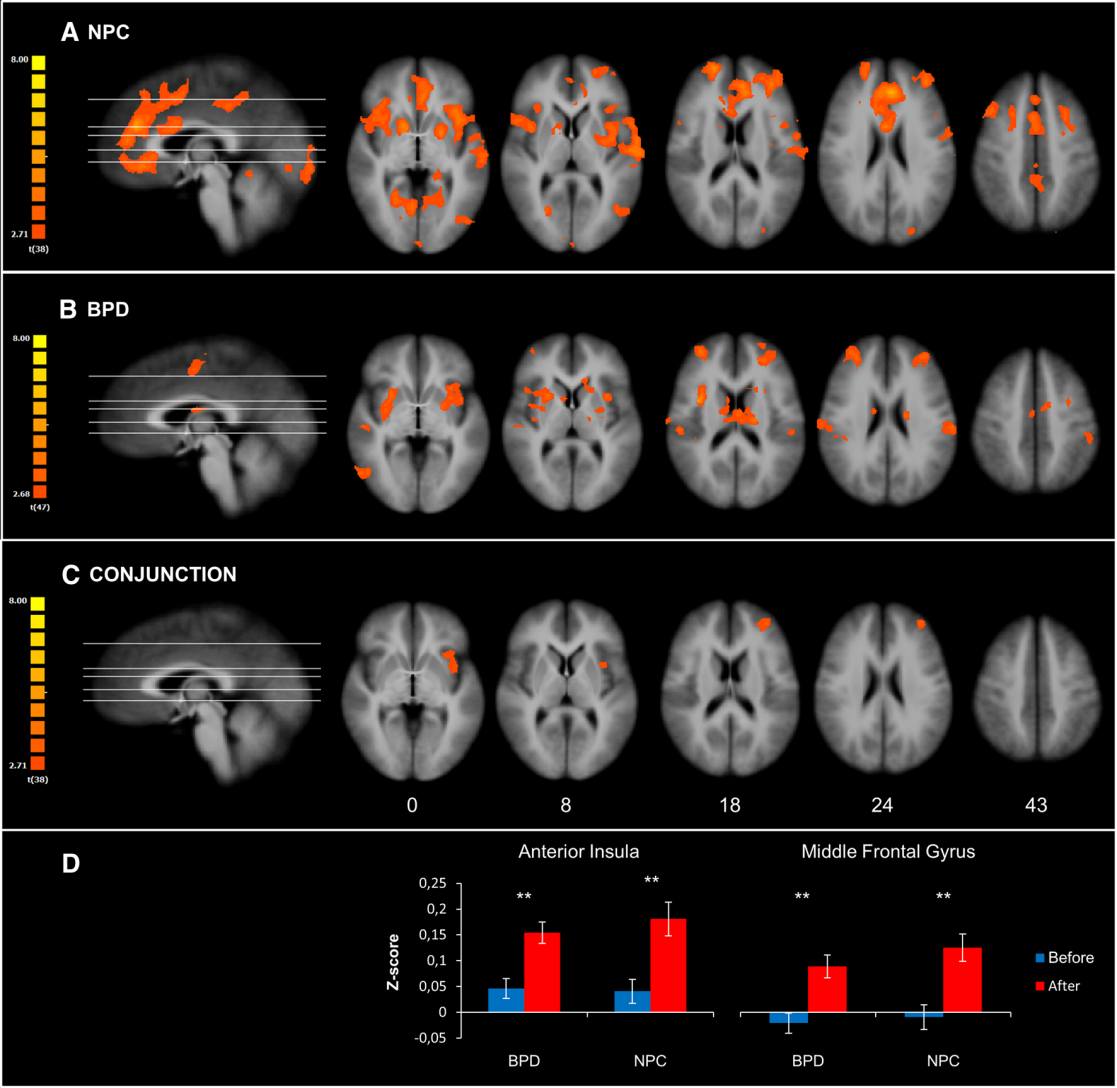
Change in the functional connectivity after the emotion regulation task. **a** Depicts the change in the amygdala resting-state functional connectivity in the NPC group, whereas **b** depicts the change in BPD patients. **c** Depicts the results of the conjunction analysis to indicate a common network of the change in the amygdala resting-state functional connectivity for both groups. **d** Depicts bar plots of the mean z-score (±SEM) of the clusters resulting from the conjunction analysis. The t-maps were corrected at the cluster level (*p* = 0.05; *k* = 1134, 891, 837 mm^3^ for the map of NPC, BPD, and conjunction, respectively) and overlaid on an anatomical image averaged over all participants in the Talairach standard space, according to the radiological convention. The hot colors indicate increased resting-state functional connectivity of the amygdala after the task. Numbers in the bottom row indicate z-coordinates of the axial sections in the Talairach standard space

After the emotion regulation task, NPC exhibited increased amygdala rsFC with the following areas: bilateral insula, striatum, superior frontal gyrus, middle frontal gyrus, ACC, lingual gyrus, right posterior cingulate cortex (PCC), medial PFC, posterior part of the left middle temporal gyrus, right precentral gyrus, right fusiform gyrus, and left cuneus (Fig. 2a; Table 2).

**Table 2:** Change in the functional connectivity after the emotion regulation task

Region	L/R	BA	Peak voxel coordinates (Talairach)			Size (mm^3^)	*t*
			*x*	*y*	*z*		
NPC							
Caudate	R	–	11	7	0	10,394	4.79
Precentral gyrus	R	9	44	19	39	1932	3.87
Superior frontal gyrus	R	10	20	58	18	3068	4.81
Fusiform gyrus	R	37	29	−50	−9	9590	4.97
Medial frontal gyrus	R	32	20	10	45	2483	3.69
Anterior cingulate gyrus	L	32	−4	37	24	21,876	6.34
Posterior cingulate gyrus	R	31	5	−35	39	3282	4.42
Lingual gyrus	L	18	−1	−92	−6	1279	4.13
Culmen	L	–	−10	−50	−9	10,382	5.22
Cuneus	L	19	−25	−86	33	1395	4.40
Middle frontal gyrus	L	10	−37	43	21	5195	4.43
Insula	L	13	−37	10	3	18,351	5.10
Middle frontal gyrus	L	8	−25	16	39	4776	4.72
Middle temporal gyrus	L	37	−43	−65	9	1682	3.88
BPD							
Superior temporal sulcus	R	22	44	−26	−6	3661	4.55
Middle temporal gyrus	R	37	47	−62	0	1269	3.79
Middle frontal gyrus	R	10	32	49	18	4197	4.15
Insula	R	13	35	4	19	6486	5.44
Caudate	L	–	−20	−14	21	2932	5.04
Supplementary motor area	R	6	2	−8	48	1679	3.83
Insula	L	13	−31	10	12	6027	5.31
Superior parietal lobule	L	7	−19	−56	60	2429	4.43
Sub-gyral	L	6	−25	−2	57	1081	3.66
Middle frontal gyrus	L	10	−31	46	21	2665	3.93
Inferior parietal lobule	L	40	−52	−32	36	1160	3.99
Postcentral gyrus	L	2	−61	−20	24	1029	3.89
Conjunction							
Middle frontal gyrus	L	10	−34	46	21	1238	3.76
Insula	L	13	−34	7	6	1232	3.48

Statistical maps (resting-state after vs. before the task) are corrected at the cluster level (*p* = 0.05; *k* = 1134, 891, 837 mm^3^ for the NPC, BPD, and conjunction map, respectively). Anatomical labels of the peak voxel coordinates are identified with the “nearest gray matter” option in the Talairach Client (www.talairach.org) [50]. *BA* Brodmann area; *R* right hemisphere; *L* left hemisphere

In BPD patients, we observed increased amygdala rsFC after the emotion regulation task with the following areas: bilateral insula, left caudate, bilateral middle frontal gyrus, posterior part of the right middle temporal gyrus, left postcentral gyrus, right supplementary motor area, and left superior and inferior parietal lobule (Fig. 2b; Table 2). The time (before vs. after) × medication (medicated vs. non-medicated) interaction within this network did not yield significant results. None of the identified clusters overlapped with the cluster indicating baseline differences between BPD and NPC, which did not significantly change after the task (*p* > 0.06, uncorrected).

The conjunction analysis showed similar increases after versus before the emotion regulation task in the amygdala rsFC with the left middle frontal gyrus and left anterior insula in both groups (Fig. 2c, d; Table 2).

These results indicate that the emotion regulation task induced changes in the pattern of amygdala rsFC in both groups, and a common network of post-task changes comprised increased amygdala rsFC with the middle frontal gyrus and anterior insula.

### Change in functional connectivity in BPD and NPC after the emotion regulation task

**Fig. 3:**
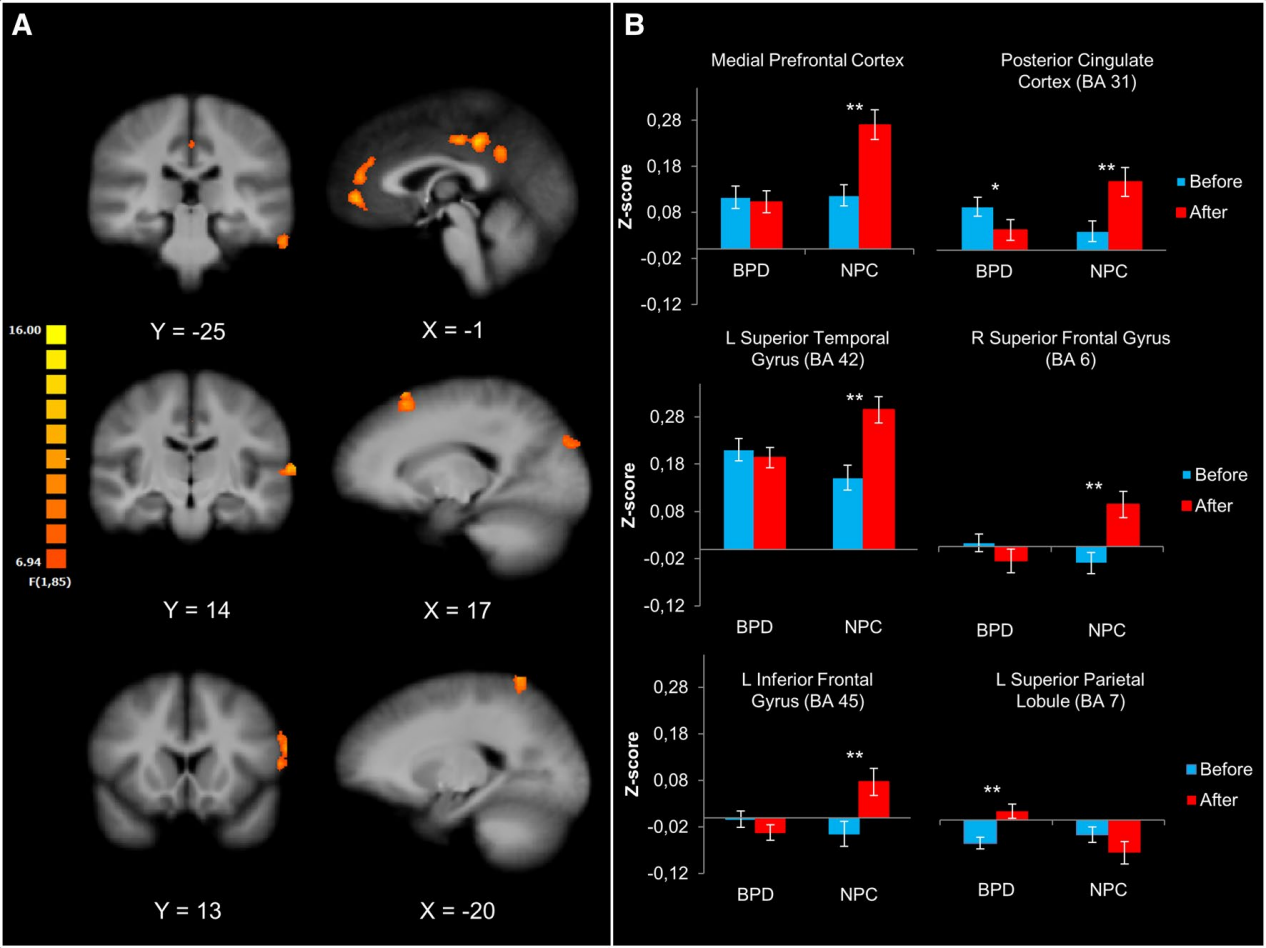
Changes in the functional connectivity in BPD and NPC after the emotion regulation task. **a** Depicts the F-map of the time × group interaction (corrected at the cluster level, *p* = 0.05; *k* = 459 mm^3^) and overlaid on an anatomical image averaged over all participants in the Talairach standard space, according to the radiological convention. **b** Depicts the bar plots of the mean z-scores (±SEM) in each group before and after the emotion regulation task and indicates significant within-group difference. The z-scores were extracted from six out of eight significant clusters (see “Methods and materials”). The between-group comparisons after the task were significant in all clusters (*p*s < 0.01), and no group differences were shown before the task

The resulting F-map revealed a significant group × time interaction in the following clusters: the right superior and left inferior frontal gyrus, left superior and inferior temporal gyrus, medial PFC, left PCC, right cuneus, and left superior parietal lobule (Fig. 3a; Table 3). The clusters of the cuneus and inferior temporal gyrus were not analyzed further, because they did not show overlap with the t-map of either BPD or NPC.

**Table 3:** Differential changes in the functional connectivity between BPD and NPC after the emotion regulation task

Region	L/R	BA	Peak voxel coordinates (Talairach)			Size (mm^3^)	*F*
			*x*	*y*	*z*		
Superior frontal gyrus	R	6	17	22	60	748	11.87
Cuneus	R	19	20	−83	30	662	10.63
(Posterior) Cingulate gyrus	L	31	−1	−32	39	2650	14.71
Medial prefrontal gyrus	R	–	2	49	3	2271	14.67
Superior parietal lobule	L	7	−16	−56	63	1290	15.08
Inferior frontal gyrus	L	45	−58	13	21	481	11.64
Superior temporal gyrus	L	42	−67	−13	9	544	12.74
Inferior temporal gyrus	L	20	−61	−23	−24	484	12.97

Statistical maps are corrected at the cluster level (*p* = 0.05; *k* = 459 mm^3^). Anatomical labels of the peak voxel coordinates are identified with the “nearest gray matter” option in the Talairach Client (www.talairach.org) [50]. *BA* Brodmann area; *R* right hemisphere; *L* left hemisphere

Simple effects on the z-scores extracted from the clusters indicated specific group differences in the change of the amygdala rsFC after the task (Fig. 3b). After the task, NPC showed an increase in the amygdala rsFC with all clusters except for the left superior parietal lobule. In contrast to NPC, BPD patients exhibited decreased post-task amygdala rsFC with PCC and increased rsFC with the left superior parietal lobule. None of these clusters overlapped with the cluster indicating baseline differences between BPD and NPC, of which the nonsignificant interaction effect was confirmed in the post hoc analysis (*p* > 0.69, uncorrected). No significant time × medication interaction within the BPD group was observed for any of the clusters (*p* > 0.06, uncorrected). Taken together, these results indicate that BPD patients and NPC showed different post-task patterns of the amygdala rsFC.

### Change in the functional connectivity and BPD characteristics

**Fig. 4:**
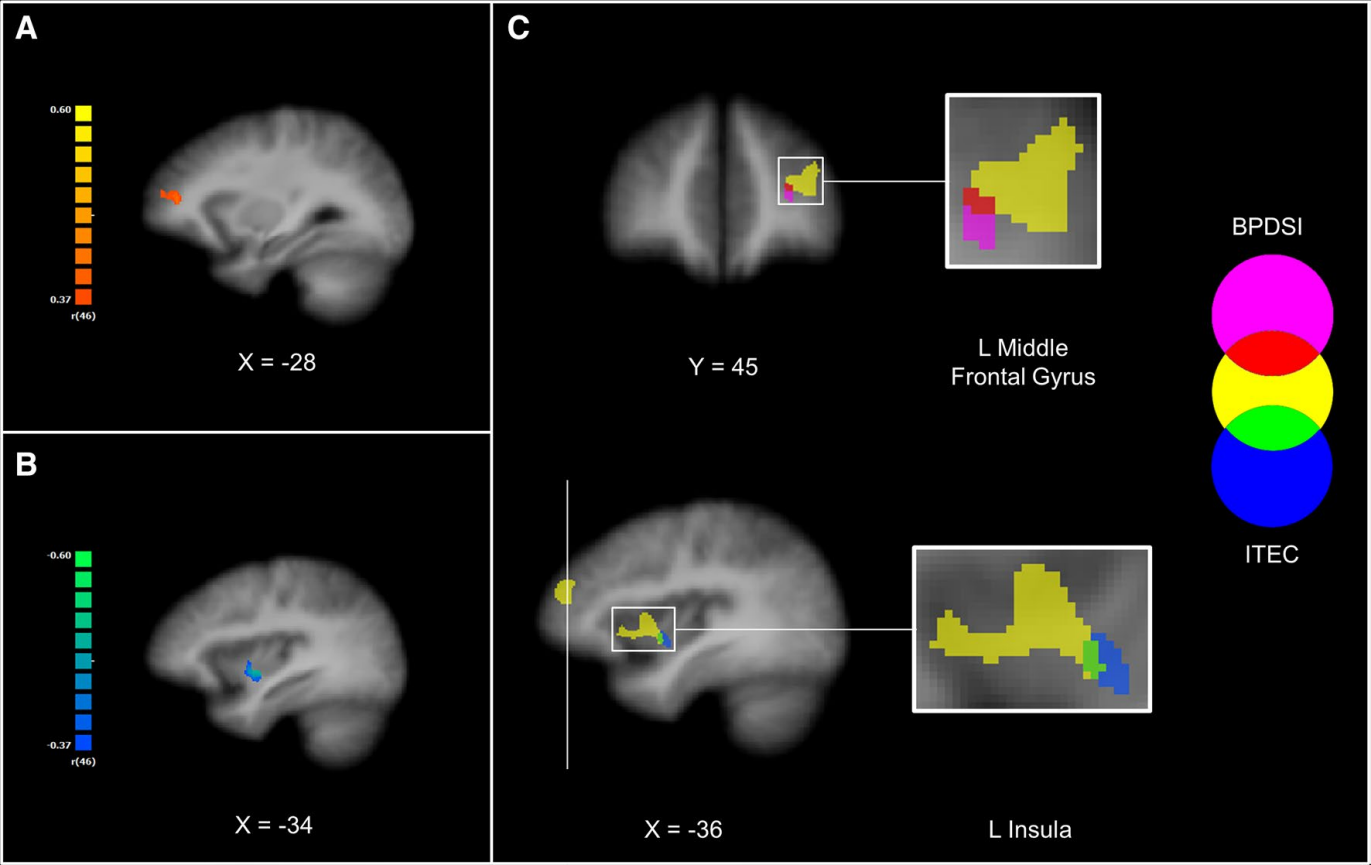
Correlational analyses of the change in the functional connectivity and BPD characteristics. **a** Depicts the correlation analysis between the magnitude of change in the amygdala resting-state functional connectivity after the task and the severity of the BPD psychopathology (BPDSI). **b** Depicts the correlation analysis with the severity of the reported childhood traumatic events (ITEC). The r-maps were corrected at the cluster level (*p* = 0.05; *k* = 270 and 351 mm^3^ for the map of BPDSI and ITEC, respectively) and restricted to the mask of the network that showed significantly increased resting-state functional connectivity of the amygdala after the task in the BPD group (see “Methods and materials”). Positive association is shown in hot colors, whereas cold colors indicate negative association. **c** The two r-maps were overlaid with the map of the conjunction analysis (see “Methods and materials”), which is depicted in yellow. Statistical maps were overlaid on an anatomical image averaged over all participants in the Talairach standard space, according to the radiological convention

The correlation analysis did not show significant associations between the severity of BPD pathology, including baseline level of dissociation, and any of the clusters retrieved from the group × time interaction. Subsequently, we tested the hypothesis that BPD characteristics are not associated with changes in the network of differential effects between BPD and NPC, but rather with the network that showed a putative response for BPD patients. Therefore, we performed a whole-brain correlation analysis with the mask of the network that showed significantly increased post-task amygdala rsFC in BPD patients. Again, we did not observe significant results for the BSI, BPD checklist or baseline level of dissociation. We, however, observed a positive association between BPDSI and the magnitude of change in the amygdala rsFC with a cluster corresponding to the left middle frontal gyrus [*r*(46) = 0.45, *p* = 0.001; peak coordinates Tal: −25, 43, 18; Fig. 4a]. We repeated the same analytical strategy for the correlation analysis with the ITEC. Again, no significant association was found between ITEC and any of the clusters of the group × time interaction. When using the mask of post-task amygdala rsFC network observed in BPD, we found a negative association of the ITEC with the magnitude of change in the amygdala rsFC with the ventral part of the left insula [*r*(46) = −0.52, *p* < 0.001; peak coordinates Tal: −34, −2, −2; Fig. 4b].

The two clusters showed minimal overlap with the clusters of the left middle frontal gyrus and the left insula that were retrieved from our conjunction analysis representing a common network of post-task changes of the BPD and NPC (Fig. 4c). These findings suggest that the severity of BPD psychopathology and childhood traumatic are associated with the changes of the intrinsic amygdala connectivity that are putative for BPD but not for NPC.

Increase in the level of dissociation in BPD patients after versus before scanning was positively associated with the change of coupling between the amygdala and two clusters retrieved from the interaction: inferior frontal gyrus (*r* = 0.34, *p* = 0.025) and superior temporal gyrus (*r* = 0.33, *p* = 0.030), demonstrating in BPD stronger amygdala rsFC with more change in dissociation. However, these results did not survive Bonferroni correction. Hence, similarly to the previous analyses, we performed whole-brain analysis with the mask of post-task amygdala rsFC network observed in BPD. We did not observe any significant results. These findings suggest that in BPD patients increased level of dissociation after scanning does not significantly contribute to their altered change of amygdala rsFC.

### Diagnosis specificity: comparison with cluster-C personality disorders patients

From the behavioral measures, we observed a weak trend toward a significant difference between the passive viewing and regulation condition across stimulus categories in CPD patients (M = 11.74, SD = 28.46, *t*(20) = 1.89, *p* = 0.07). There was no significant difference between CPD and BPD (*p* = 0.98) as well as NPC (*p* = 0.63) in the difference between the passive viewing and regulation condition, which suggests that the difference score is similar in magnitude and failed to reach significance in CPD likely due to smaller number of participants. The amygdala rsFC network observed in CPD before and after the task was similar to the patterns observed in the BPD and NPC (Fig. S3b). The interaction analysis between BPD and CPD patients did not yield significant results after Bonferroni correction. We additionally performed a conjunction analysis of the whole-brain F-maps indicating the interaction effects BPD versus NPC and CPD versus NPC to explore whether there were common altered changes in the amygdala rsFC in both patients groups. Again, no significant results were observed. However, the uncorrected map (thresholded at *p* = 0.01) showed a cluster in the left vmPFC (peak coordinates Tal: −4, 43, −3). We used the z-scores extracted from the cluster in the subsequent post hoc analysis, using two-way mixed ANOVA with three groups and observed a significant time × group interaction (*p* = 0.001). Only NPC exhibited a significant post-task increase in the amygdala rsFC with the cluster [*t*(38) = 3.86; *p* < 0.001]. The exploratory results suggest that effortful emotion regulation leads to an altered amygdala rsFC with the vmPFC that might be common for personality disorders characterized by emotional problems.

## Discussion

In the present study, we investigated whether a cognitive emotion regulation task induces different changes of the amygdala rsFC with the PFC in BPD patients, compared to NPC. Although according to the behavioral results both groups reported similar change in their emotional state due to cognitive regulation, BPD patients exhibited a different post-task amygdala rsFC, which did not involve the brain network of pre-task differences between the groups. While NPC showed increased post-task amygdala rsFC with the medial, dorsolateral and ventrolateral PFC, and superior temporal gyrus, BPD patients exhibited a lack of change in this network. Compared to NPC, BPD patients surprisingly showed decreased post-task amygdala rsFC with the PCC and increased rsFC with the superior parietal lobule.

Effortful emotion regulation is a complex cognitive process that involves neuronal systems responsible for emotion generation and regulation [29, 51]. The amygdala is a key node detecting potential threats [52, 53], and together with the striatum, anterior insula and dACC forms a salience network [15], which selects stimuli as relevant for current goals and thus initiates subsequent regulatory mechanisms subserved by a fronto-parietal cognitive control network [54]. We observed in NPC that our emotion regulation task induced changes in the amygdala rsFC patterns that involved both the emotion generating and regulatory networks. Hence, we infer that such enduring changes reflect employment of neuronal circuits, which subserve the complete process of effortful emotion regulation including the generation (i.e., effects of passive viewing or presentation of different stimulus categories) and regulation of emotions, as these components could not be strictly disentangled.

In light of this, BPD patients seem to modulate their emotions with different brain networks. They showed altered post-task amygdala rsFC with the dlPFC, vlPFC, and temporal cortex that comprise the cognitive control network used to maintain, select, evaluate, and reinterpret the emotion-related representations retrieved from the lateral temporal cortex [27, 29]. Moreover, when BPD patients are confronted with the effortful emotion regulation task, they exhibit additional post-task cross-talk between the amygdala and superior parietal lobule, which is implicated in attention [55, 56] and episodic memory retrieval [57]. Additionally, the more severe the condition of BPD according to the BPDSI, the more areas of the left middle frontal gyrus within the post-task intrinsic amygdala network was observed. We speculate that severely affected BPD patients engaged more the middle frontal gyrus to maintain their reappraisals in working memory [27, 28] due to following reasons. Over the course of the task, the passive viewing condition can induce (un-)intentional regulation in patients who are emotionally hyperreactive [e.g., 33, 58, 59] and/or the regulation cannot be terminated by the impaired inhibitory loop, which involves the vlPFC, when a goal-appropriate reappraisal has been selected [60]. To overcome this deficit, BPD patients might employ a compensatory but maladaptive strategy, such as dissociation, which typically occur due to overwhelming emotions in stressful situations [61]. While previous research shows that dissociation is associated with rsFC and predicts changes in brain activity due to emotional tasks [13, 14, 19], in the current study, we did not find evidence for similar associations. Yet, these associations might become apparent when BPD patients encounter emotional challenge. To this end, we additionally explored whether increased dissociation after scanning is associated with differential change of the rsFC in BPD. Although this additional analysis did not survive correction for multiple comparisons, it is noteworthy for future studies to report that increased dissociation was moderately associated with increased amygdala rsFC with the inferior frontal gyrus and superior temporal gyrus. While these findings might potentially indicate a tendency for a compensatory mechanism in BPD, they are speculative and should be taken with caution.

It is assumed that the cognitive control network of effortful emotion regulation modulates the amygdala activity indirectly via the vmPFC [29, 30, 62]. The amygdala is extensively interconnected with the mPFC [63], and its FC strength predicts lower levels of anxiety and effective emotion regulation [64]. Previously, Kamphausen et al. [11] reported that an exaggerated amygdala response to threat in BPD patients is associated with a failure of the regulatory amygdala–mPFC connectivity loop. Similarly, the present results show that enduring effects of emotion regulation in BPD are absent in the amygdala–mPFC circuit. The plausible dysfunction of this network may underlie impaired cognitive emotion regulation in BPD patients. Although the altered neurocognitive component of effortful emotion regulation might be specific for BPD [16], our results suggest that the direct regulation of the amygdala by vmPFC seems to be common for other personality disorders characterized by emotional problems.

Effortful emotion regulation induced altered post-task changes of the intrinsic amygdala connectivity with the PCC, which we did not expect. The PCC is a key node in the default mode network (DMN) and has been implicated in self-referential processing and autobiographical memory [65, 66]. Veer et al. [24] reported increased amygdala rsFC with PCC and the adjacent precuneus after acute social stress in NPC, which after the task might promote the evaluation of emotionally salient events stored in autobiographical memory to prepare for future challenges. Individuals who experienced childhood maltreatment may not be able to benefit from such an adaptive strategy [67]. Childhood abuse and neglect is prevalent in BPD patients [68] and contributes to their emotion dysregulation [69]. BPD patients also show evidence for altered self-referential processing at rest [13, 19] and during pain, which is associated with dissociation [70], likely influenced by childhood traumatization [71]. Although we did not find the severity of early-life abuse and neglect in BPD patients to be associated with post-task changes in the amygdala rsFC with the PCC, a negative association was observed in the left insula, a brain region implicated in somatosensory processing and interoception [72] and responsible for switching between the DMN and fronto-parietal control network [73]. Hence, we speculate that BPD patients tend to avoid self-related appraisals of salient emotional stimuli, which might be partly mediated by their blunted affect caused by childhood maltreatment. Future studies could directly investigate brain connectivity of the intertwined relations between emotion, autobiographical memory, and childhood abuse and neglect in BPD [cf., 74, 75].

### Limitations

In the current study, we included only females, which hamper generalizability to males. Second, the BPD patients represented a rather heterogeneous group given the presence of co-occurring disorders, with most prominent depression and substance abuse. Comorbid Axis I disorders are typical in BPD, and BPD patients without Axis I co-occurring disorders are rare and not representative for the disorder. As a consequence, we cannot exclude the possibility that our results might be affected by these comorbidities. Furthermore, left-handed participants were included in the study. A third limitation of our study pertains to the possible impact of medication intake, which might have influenced the intrinsic organization of connectivity networks [76]. However, to recruit a representative and severe clinical sample we did not exclude patients on medication. We performed additional analyses within the BPD group, medicated versus non-medicated, to exclude acute effects of medication on our results, as adding medication as a covariate could remove some substantial part of the variance associated with group differences. Fourth, we have reported all results at a relatively lenient initial threshold of *p* < 0.01 and corrected for multiple comparisons at cluster level at *p* < 0.05. However, to limit the possibility of false positives we restricted the interpretation of only those interaction effects that were based on an effect of the task in at least one group regardless potential group differences. Finally, we should always be cautious about the interpretation of the amygdala FC, because the blood-oxygenation level-dependent signal of the amygdala is susceptible to physiological confounds due to its proximity to draining veins. Of note, the results of the correlation analyses should be interpreted with some caution, because of a small sample size.

## Conclusion

In BPD patients, the emotion regulation task failed to increase amygdala intrinsic FC with brain regions essential for effortful emotion regulation, which suggests: (a) altered cognitive control typically used to indirectly alleviate distress by reinterpreting the meaning of emotional stimuli; (b) impaired direct regulation of emotional responses, which might be common for personality disorders; (c) avoidance of self-related appraisals induced by social emotional stimuli. The lack of enduring post-task effects in BPD patients in these networks might hamper their cognitive control over subsequent emotional challenge. These findings portray a complex picture and vicious circle of emotion dysregulation in BPD.

## Electronic supplementary material

Below is the link to the electronic supplementary material.

Supplementary material 1 (DOCX 1190 kb)
