# Supplementary material for: Deficient amygdala–prefrontal intrinsic connectivity after effortful emotion regulation in borderline personality disorder
Source: Eur Arch Psychiatry Clin Neurosci. 2016 Dec 30;267(6):551–65. doi: 10.1007/s00406-016-0760-z (PMC5561271; doi:10.1007/s00406-016-0760-z)
Supplement: Supplementary file 1 — Supplementary material 1 (DOCX 1190 kb) [file 406_2016_760_MOESM1_ESM.docx]

**Supplementary Materials**

*European Archives of Psychiatry and Clinical Neuroscience*

**Deficient amygdala-prefrontal intrinsic connectivity after effortful emotion regulation in borderline personality disorder**

Blazej M. Baczkowski, Linda van Zutphen*, Nicolette Siep, Gitta A. Jacob, Gregor Domes, Simon Maier, Andreas Sprenger, Alena Senft, Bastian Willenborg, Oliver Tüscher, Arnoud Arntz, & Vincent van de Ven

^*^ Corresponding author

Dept. of Clinical Psychological Science, Faculty of Psychology and Neuroscience, Maastricht University, P.O. Box 616, 6200 MD, Maastricht, The Netherlands

Email: [linda.vanzutphen@maastrichtuniversity.nl](mailto:linda.vanzutphen@maastrichtuniversity.nl)

**Supplementary Materials**

**Methods and Materials**

The text below is almost identical to van Zutphen et al, [submitted] because it is a part of a larger study performed by the same research group.

**Participants**

Borderline personality disorder (BPD) and Cluster-C personality disorder (CPD) patients were recruited from the mental health clinics at local sites: the Virenze-Riagg in Maastricht (the Netherlands), PsyQ in Heerlen (the Netherlands), the borderline treatment unit of the Department of Psychiatry and Psychotherapy at the Medical Center Freiburg (Germany), the Department of Psychiatry and Psychotherapy at the University Hospital Lübeck (Germany) and the Institute for Behavior Therapy Training Hamburg (Germany). BPD patients were taking part in an international multicenter RCT on group schema therapy for BPD [[for detailed description, 1](#_ENREF_1)] and their measurements reported in this study were collected before the start of their therapy, unless impossible due to scheduling problems (scanning only) in which case the fMRI-measurements had to be finished within three months from the start of the treatment. Only females were chosen since gender might influence emotional processing [[2](#_ENREF_2)], and because in mental health care borderline personality disorder is more often diagnosed in females. We excluded homosexual females, because we used heterosexual erotic stimuli. General exclusion criteria were lifetime psychotic or bipolar disorder type-I, attention-deficit/hyperactivity disorder, dissociative identity disorder, serious and/or unstable medical illness, substance dependence needing clinical detoxification and fMRI exclusion criteria (i.e. claustrophobia, metal objects, cardiac arrhythmia, epilepsy, tattoos at neck/head and pregnancy).

BPD and CPD patients were diagnosed according to the DSM-IV criteria, using the Structural Clinical Interview (SCID) I [[3](#_ENREF_3)] and II [[4](#_ENREF_4)], assessed by trained interviewers. BPD patients were further screened with the BPD Severity Index [[5-7](#_ENREF_5)], of which the score > 20 indicates an additional inclusion criterion for BPD [[1](#_ENREF_1)]. Narcissistic and antisocial personality disorder (PD), full or sub-threshold, were excluded for reasons related to the clinical trial in which the study sample participated [[1](#_ENREF_1)]. CPD patients were not allowed to score full or sub-threshold cluster-B PD, and > 2 BPD criteria. NPC did not meet DSM-IV criteria for any axis I or axis II disorder, as assessed by SCID-screeners [[3](#_ENREF_3), [4](#_ENREF_4)]. Positive items on screeners were checked with SCID interviews. Additional assessments involved the Brief Symptom Inventory [[8](#_ENREF_8)], BPD checklist [[9](#_ENREF_9)] and Interview for Trauma Events in Childhood [[10](#_ENREF_10)].

Eight participants were excluded because of invalid, incomplete or unavailable data (3 BPD, 2 NPC, 3 CPD), five because of excessive head movement of more than 4 mm (2 BPD, 2 NPC, 1 CPD), two NPC had scores above .70 on the BSI and three CPD patients had scores above 100 on the BPD checklist. Eight participants (6 BPD, 1 NPC, 1 CPD) explicitly reported in the exit questionnaire that they fell asleep during any of the resting state runs, and therefore were excluded. Three BPD and two CPD subjects were excluded because they had an IQ outside the range of 70-120. Additionally, two NPC with IQ scores above the 95-percentile were excluded to guarantee that NPC were matched to the BPD participants.

**Measures**

*Borderline Personality Disorder Severity Index (BPDSI-VI)* - The BPDSI is a semi-structured clinical interview assessing frequency and severity of borderline manifestations [[5-7](#_ENREF_5)]. The 70-items reflect the nine borderline criteria described in the DSM-IV. For each item the frequency of the last three months is rated on an 11-point scale, ranging from 0 (never) to 10 (daily). The scores on the subscales provide information on the severity of each of the DSM-IV dimensions, derived by averaging the items scores. The total score is the sum of the nine dimensions scores, ranging from 0 to 90, with an internal consistency of Cronbach’s α = 0.93 and subscales ranged between 0.41 and 0.83 [[5](#_ENREF_5)]. A total score of 20 distinguishes borderline personality disorder from other personality disorders [[6](#_ENREF_6)].

*Brief Symptom Inventory (BSI)* - The Brief Symptom Inventory is a brief psychological self-report inventory of general symptoms of psychopathology during the past week [[8](#_ENREF_8)]. It is a short alternative for the Symptom Checklist-90-R from which it was developed. It contains 53 items divided over nine dimensions: somatization, obsession-compulsion, interpersonal sensitivity, depressive mood, anxiety, hostility, phobic anxiety, paranoid ideation and psychoticism. Answers are scored on a 5-point Likert scale, ranging from 0 (not at all) to 4 (extremely). Scores of the dimensions are calculated by summing the values for the items divided by the number of items within the subscales. The total score measures the level of symptomatology, which is the sum of the nine dimensions plus the four additional items divided by total number of items. The internal consistency showed a Cronbach’s α of 0.96 for the total instrument and ranged between 0.71 and 0.85 for its subscales [[8](#_ENREF_8), [11](#_ENREF_11)]. To distinguish patients from non-patients a cutoff score of 0.70 is suggested [[12](#_ENREF_12)].

*BPD Checklist* - The BPD Checklist is a self-report questionnaire used to assess the burden of borderline symptoms as experienced during the last month [[9](#_ENREF_9)]. It consists of 47 items based on the nine dimensions of borderline personality disorder in DSM-IV. Items must be rated on 5-point Likert scale, ranging from 1 (not at all) to 5 (extremely). Next to the total score, also the scores for the nine subscales can be calculated. Scores above 100 signify borderline pathology and therefore is indicative as cutoff for inclusion criteria. When control patients showed an elevated score, an extra check with the SCID-II borderline section was done.

*Interview for Traumatic Events in Childhood (ITEC) -* The Interview for Traumatic Events in Childhood is a retrospective, semi-structured interview to measure childhood maltreatment prior the age of 18 including sexual (12 items), physical (13 items) and emotional abuse (9 items) and emotional (6 items) and physical neglect (15 items) [[10](#_ENREF_10)]. For each item the participant experienced maltreatment, follow-up questions are used to gather more detailed information about the perpetrator(s), age of onset, frequency, duration of the trauma and the impact on the victim in the past and in the present. This information was used to calculate a severity score between 0 and 1, such that the score increased with the severity of the event itself, the closeness of the perpetrator, the younger age of onset, the longer duration of the event, and the higher impact on the victim. For each subscales the severity scores for the events are summed, the higher this score, the more severe the maltreatment. Internal consistencies of these scales were moderate to excellent, with Cronbach’s α varying between 0.58 and 0.89 with a mean of 0.79 [[10](#_ENREF_10)]. In addition to the victimization scales, similar scales were created for witnessing the various forms of maltreatment. In current study only the victim scales were reported. On average the administration time is about 30 minutes but can take up to one hour in case of multiple maltreatments.

*Wechsler Adult Intelligence Scale* - IQ was estimated by means of four subtests of the Wechsler Adult Intelligence Scale, including two verbal (i.e. Vocabulary and Similarities) and two nonverbal tests (i.e. Block design and Matrix reasoning). Together these subtests correlate strongly with general intellectual ability [[13](#_ENREF_13)]. IQ was estimated based on the optimized regression equation: 39.05 + (1.54*comprehension score) + (1.64*matrix reasoning score) + (1.48*similarities score) + (0.98*picture arrangement score). If the WAIS score was not available (borderline patients *n* = 11, non-patients *n* = 3 and control patients *n* = 1) an estimation of the IQ was made based on the education level, using the regression equation from our present sample per group; IQ borderline patients = 85.932 + (3.360*ISCED code), IQ non-patients = 85.996 + (3.659*ISCED code) and IQ control patients = 81.529 + (4.226*ISCED code). These regression equations explained respectively 22%, 31% and 36% of the variance in IQ. Level of education of both the Dutch and German educational systems were transformed into the International Standard Classification of Education (ISCED).

*Dissociation and Anxiety* - Present state dissociative experiences were assessed using four items of the Dissociation-Tension-Scale [[14](#_ENREF_14)], containing somatic dissociation such as changes in perception of one’s body, hearing, vision and pain. Additionally two items concerning the level of anxiety and the level of nervousness were added. Responses were indicated on a visual analogue scale, ranging from 0 not at all to 100 extreme. The dissociation score was the averaged across the four dissociation items. Internal consistencies of the dissociation score proved to be good in current sample, with a Cronbach’s α of 0.83 concerning dissociation before scanning and a Cronbach’s α of 0.88 concerning dissociation after scanning.

*Self-Assessment Manikin Scale* - The pictures shown during the scanning session were qualitatively assessed for valence and arousal using the Self-Assessment Manikin Scale. The Self-Assessment Manikin Scale consists of a series of human-like figures to measures the affective reaction of a person to stimuli [[15](#_ENREF_15)]. Intensity of valence and arousal were both rated on a 9-point scale, with for valence 1 being extremely unpleasant and 9 being extremely pleasant, and for arousal 1 being most calm and 9 being most aroused.

**MRI data acquisition**

Structural and functional MRI data were acquired with 3 T scanners at three sites: in Maastricht on a Siemens Magnetom Allegra head-only scanner equipped with a birdcage headcoil (Siemens Medical Systems, Erlangen, Germany), in Freiburg on a Siemens tim-Trio Magnetom whole body scanner (Siemens Medical Systems, Erlangen, Germany) equipped with an 8-channel headcoil, and in Lübeck on a Philips Achiva whole body scanner equipped with an 8-channel headcoil (Philips Healthcare, Best, The Netherlands). The BPD patients from Heerlen were scanned in Maastricht and the BPD patients from Hamburg were scanned in Lübeck. In Maastricht 12 BPD, 9 NPC and 11 CCP were scanned, Freiburg scanned 13 BPD, 18 NPC and 6 CCP, finally 23 BPD, 12 NPC and 4 CCP were scanned in Lubeck.

**FMRI data preprocessing**

FMRI data preprocessing was carried out using BrainVoyager 2.8 (Brain Innovation, Maastricht, The Netherlands), NeuroElf (an MR imaging analysis toolbox, www.neuroelf.net) and custom routines in Matlab (Mathworks, Inc). The first two volumes were removed due to saturation effects. The following preprocessing steps were conducted: slice time correction with sinc interpolation, 3D motion correction for three translational and three rotational directions with trilinear/sinc interpolation, spatial smoothing with a 4 mm full-width-at-half-maximum isotropic Gaussian kernel, and removal of linear trend. Additionally, a temporal band-pass filter of 0.01 to 0.1 Hz was applied to reduce cardiac and respiratory noise. In order to further reduce the non-neuronal components of fMRI datasets, a component based method (CompCor) was applied to remove the significant principal components representing data artifacts from head motion, cerebrospinal fluid, and white matter [[16](#_ENREF_16)].

Spatial normalization was conducted using a high-resolution anatomical datasets. Participants underwent a second fMRI session (data presented elsewhere), in which a second anatomical scan was acquired. In order to obtain a high resolution and high contrast anatomical scan, the individual anatomical datasets from the two fMRI sessions were averaged when possible. Anatomical datasets were corrected for inhomogeneity intensity and non-brain tissue was removed. Such individual anatomical datasets were used to co-register the single subject 4D functional datasets. All single subject datasets were normalized in Talairach space (1 × 1 × 1 mm; Talairach & Tournoux, 1988).

**Supplementary Figures**

Supplementary Figure S1


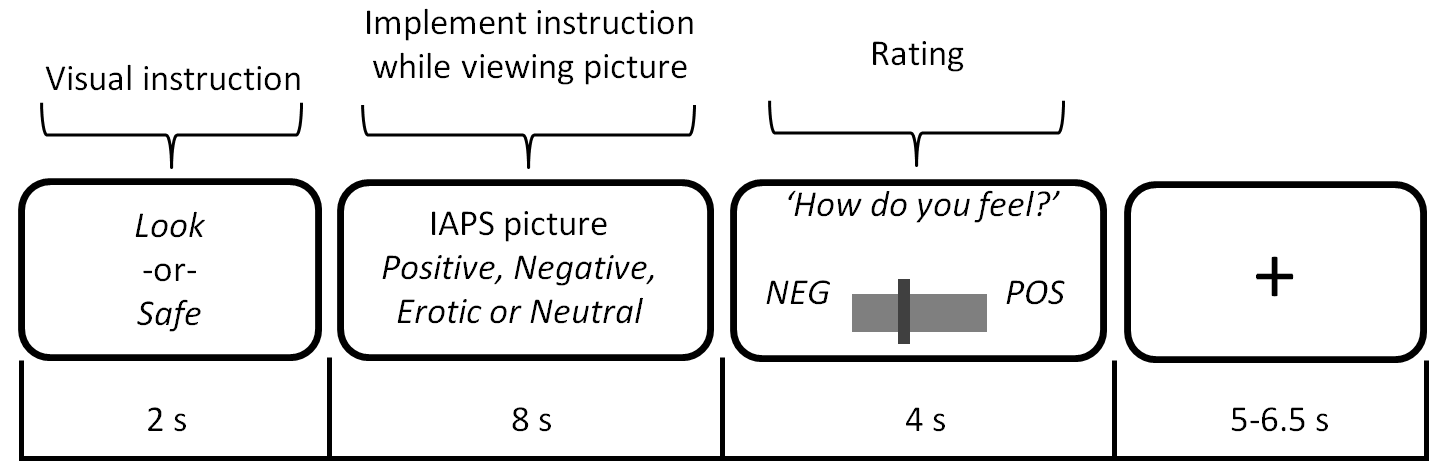


**Fig. S1** Schematic overview of a single trial of the Emotion regulation paradigm

Detailed description of the task is available elsewhere [van Zutphen et al., submitted], but for a schematic overview of a single trial see figure.

Supplementary Figure S2


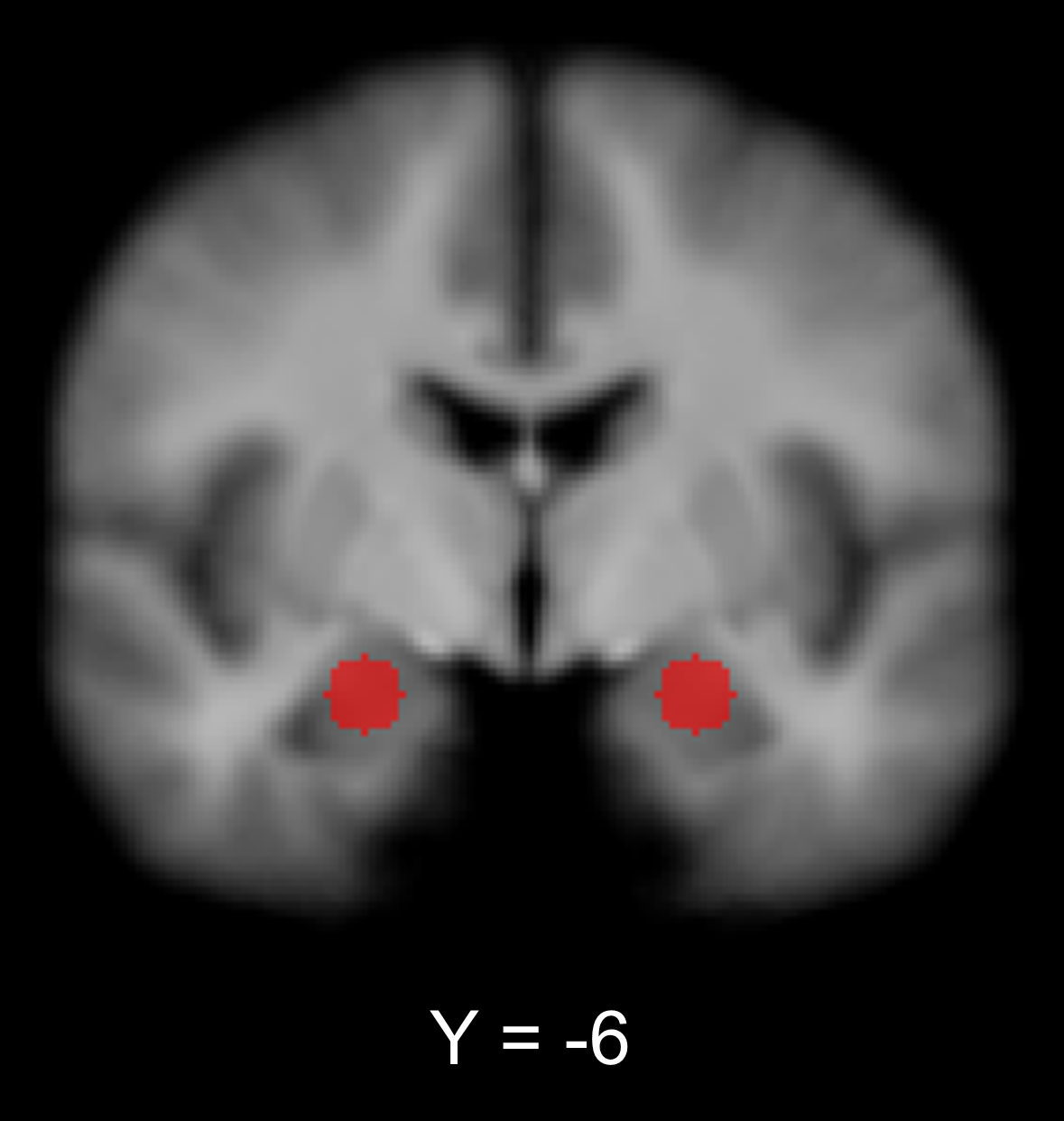


**Fig. S2** Amygdala seeds.

A 5 mm sphere of the seeds used in the functional connectivity analyses were centered around the coordinates: ±22, -6, -14 (Talairach space).

Supplementary Figure S3


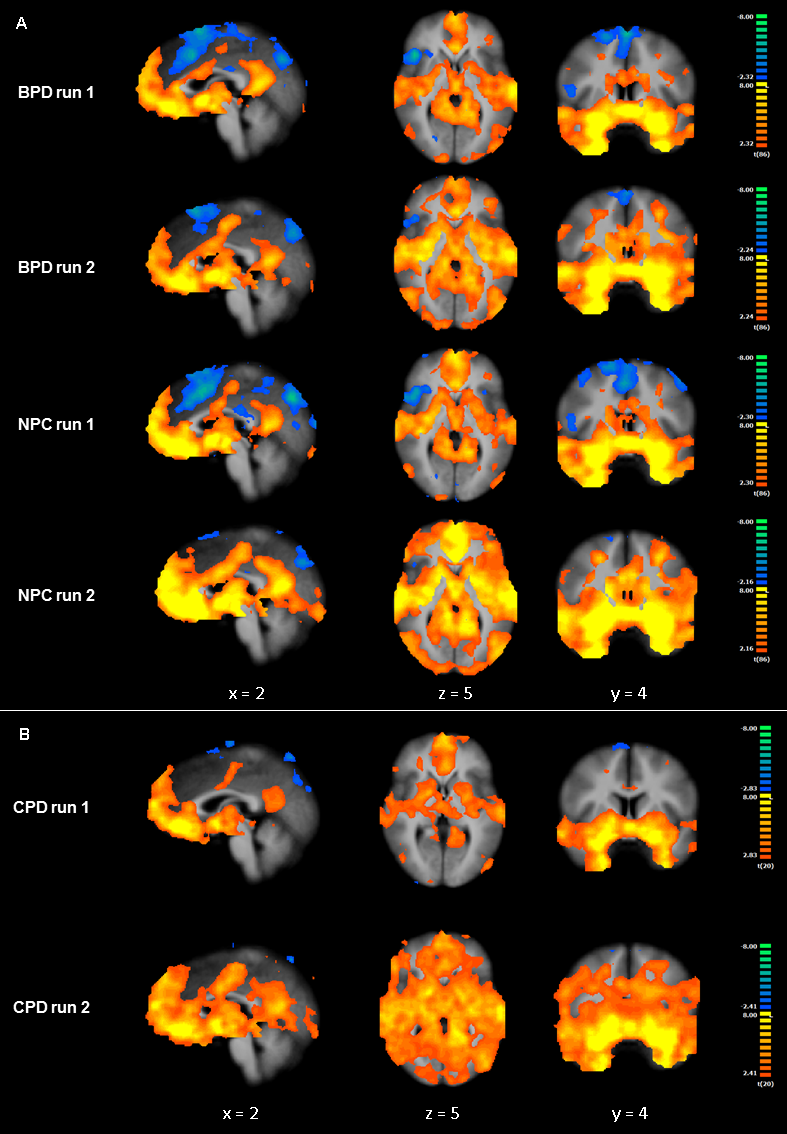


**Fig. S3** Main effects of the amygdala resting-state functional connectivity in BPD and NPC (Panel A), and CPD (Panel B).

The t-maps (whole brain FDR voxel-level corrected *p* < 0.05) depict average amygdala functional connectivity before and after the emotion regulation task. Statistical maps were overlaid on an anatomical image averaged over all participants in the Talairach standard space, according to the radiological convention. Positive connectivity is shown in hot colors whereas cold colors indicate negative connectivity. Significant voxels outside cerebrum were masked for illustration purposes.

**References**

1. Wetzelaer P, Farrell J, Evers SM, Jacob GA, Lee CW, Brand O, van Breukelen G, Fassbinder E, Fretwell H, Harper RP, Lavender A, Lockwood G, Malogiannis IA, Schweiger U, Startup H, Stevenson T, Zarbock G, Arntz A (2014) Design of an international multicentre rct on group schema therapy for borderline personality disorder. BMC Psychiatry 14:319. doi: 10.1186/s12888-014-0319-3

2. Whittle S, Yucel M, Yap MB, Allen NB (2011) Sex differences in the neural correlates of emotion: Evidence from neuroimaging. Biol Psychology 87:319-333. doi: 10.1016/j.biopsycho.2011.05.003

3. First MB, Spitzer RL, Gibbon M, Williams JBW (1994) Structured clinical interview for dsm-iv axis i disorders (scid-i). Biometric Research Department, New York

4. First MB, Spitzer RL, Gibbon M, Williams JBW, Benjamin L (1997) Structured clinical interview for dsm-iv axis ii personality disorders (scid-ii). Biometric Research Department, New York

5. Arntz A, van den Hoorn M, Cornelis J, Verheul R, van den Bosch WM, de Bie AJ (2003) Reliability and validity of the borderline personality disorder severity index. J Pers Disord 17:45-59. doi: 10.1521/pedi.17.1.45.24053

6. Giesen-Bloo JH, Wachters LM, Schouten E, Arntz A (2010) The borderline personality disorder severity index-iv: Psychometric evaluation and dimensional structure. Pers Indiv Differ 49:136-141. doi: DOI 10.1016/j.paid.2010.03.023

7. Kroger C, Vonau M, Kliem S, Roepke S, Kosfelder J, Arntz A (2013) Psychometric properties of the german version of the borderline personality disorder severity index--version iv. Psychopathology 46:396-403. doi: 10.1159/000345404

8. Derogatis LR (1993) Bsi brief symptom inventory: Administration, scoring, and procedure manual. National Computer Systems, Minneapolis, MN

9. Arntz A, Dreessen L (1995) Bpd-klachtenlijst 47 [bpd checklist]. Maastricht University, The Netherlands

10. Lobbestael J, Arntz A, Harkema-Schouten P, Bernstein D (2009) Development and psychometric evaluation of a new assessment method for childhood maltreatment experiences: The interview for traumatic events in childhood (itec). Child Abuse Negl 33:505-517. doi: 10.1016/j.chiabu.2009.03.002

11. De Beurs E, Zitman FG (2006) De brief symptom inventory (bsi): De betrouwbaarheid en validiteit van een handzaam alternatief voor de scl-90 [the brief symptom inventory (bsi): The reliability and validity of a brief alternative of the scl-90]. Maandblad Geestelijke Volksgezondheid 61:120-141.

12. De Beurs E (2004) Handleiding bij de brief symptom inventory (bsi). Pits Publishers, Leiden

13. Schrimsher GW, O'Bryant SE, O'Jile JR, Sutker PB (2008) Comparison of tetradic wais-iii short forms in predicting full scale iq scores in neuropsychiatric clinic settings. J Psychopathol Behav 30:235-240. doi: DOI 10.1007/s10862-007-9066-9

14. Stiglmayr CE, Shapiro DA, Stieglitz RD, Limberger MF, Bohus M (2001) Experience of aversive tension and dissociation in female patients with borderline personality disorder -- a controlled study. J Psychiatr Res 35:111-118. doi: 10.1016/S0022-3956(01)00012-7

15. Bradley MM, Lang PJ (1994) Measuring emotion: The self-assessment manikin and the semantic differential. J Behav Ther Exp Psychiatry 25:49-59. doi: 10.1016/0005-7916(94)90063-9

16. Behzadi Y, Restom K, Liau J, Liu TT (2007) A component based noise correction method (compcor) for bold and perfusion based fmri. Neuroimage 37:90-101. doi: 10.1016/j.neuroimage.2007.04.042
